# Maternal pesticide exposure and child neuro-development among smallholder tomato farmers in the southern corridor of Tanzania

**DOI:** 10.1186/s12889-020-10097-6

**Published:** 2021-01-20

**Authors:** Peter M. Chilipweli, Aiwerasia Vera Ngowi, Karim Manji

**Affiliations:** 1 Department of environmental health sciences, Ruaha catholic university (RUCU), P.O.BOX 774, Iringa, Tanzania; 2 Department of Environmental and Occupational Health, School of Public Health and Social Sciences, Muhimbili University of Health and Allied Sciences, P.O.BOX 65001, Dar es Salaam, Tanzania; 3 Department of Paediatrics and child Health, School of Medicine Muhimbili University of Health and Allied Sciences, P.O.BOX 65001, Dar es Salaam, Tanzania

**Keywords:** Maternal exposure, Pesticides, Child neuro-development, Southern agricultural growth corridor, Smallholder tomato farmer, Exposure scenario, Malawi child development tool

## Abstract

**Background:**

Exposure to pesticides with its associated effects prenatally and in early childhood has not received much attention. There is little scientific data on this aspect in Tanzania therefore this study was meant to contribute to the deficit in the subject.

**Method:**

A cross-sectional study was conducted to a sample of 286 participants of mother to child pair, whereby 172 and 114 were exposed and non-exposed respectively. Mothers who had been working in tomato sprayed farms were exposed and mothers who had not been working in the tomato sprayed farms were un-exposed. Child aged 0–6 years was chosen from each mother sampled but only one child found to be the youngest with the classified age was enrolled. Malawi child development Tool (M-DAT) was employed to assess the child level of development, height, and weight of the children were collected and analyzed by the WHO anthropometric calculator. A checklist and questionnaire were used to observe and assess maternal exposure. Bivariate and Multivariate analysis were conducted to assess the relationship between various factors of exposure.

**Results:**

Overall 15% of the children examined were not well developed and the most used pesticides were those posing neuro-development effects. On the bivariate analysis model, mothers who worked while pregnant were more likely to have a child with neuro-developmental effect OR=5.8(1.29–26.3). On multivariate analyses adjusted for age of the mother, variables which remain in the model were a distance from home [AOR=9.4(4.2–20.5)], and working while pregnancy [AOR=5.8(1.29–26.3)] other were removed due to collinearity effect. None of confounders had a potential significant effect but only nutrition seems to be the effect modifier [AOR=7.8(1.29–36.3)] when analyzed with working while pregnancy.

**Conclusions:**

The findings from this study have indicated that maternal pesticide exposure among farmworker residents in the SAGCOT area has a potential association with child developmental effect.

**Supplementary Information:**

The online version contains supplementary material available at 10.1186/s12889-020-10097-6.

## Background

Child neuro-development concern has received much attention as the United Nations Sustainable Development Goals (SDGs) have placed early child development on the global policy agenda goal 4.2 [1–3]. Over 30% of the global burden of disease in children is attributed to environmental factors, including pesticides [4]. Though it is necessary to use to increase crop yield and to minimize post-harvest losses [5]. Liu et al. [6] reported that, pesticides are associated with poor behavioral and neurological outcomes in young children. It is marked that 80% of children with disabilities live in developing countries [7–11].

Young children may be highly exposed to these pesticides because of their normal tendency to explore their environment orally, and their proximity to potentially contaminated floors, surfaces, and air [12, 13]. Child potential routes of exposure to pesticides include breast milk, ingestion of food contaminated with pesticides, and household exposures via dermal contact [14–20]. Maternal use of pesticides during pregnancy and on early childhood has potentially contributed to child exposure which leads to child development effects [21–23].

Various studies [24–27] have reported that women who use pesticides in their homes or yards are two times more likely to have children with neural tube defects than women who do not [18, 28–30]. But studies in the southern agricultural corridor [31] have found that water samples from the area had a large amount of organophosphate and carbamate compared to other areas and the child neuro-development effect has not been assessed. Thus exposure to pesticides at early childhood with its associated effects has not received much attention, and little scientific data is available especially in southern Tanzania where women are working on weeding and harvesting and pesticide use, and hence drifting is intensive.

Therefore, this study has addressed the gap by first determine the magnitude of child development effect among women in the SAGCOT (Southern Agricultural Growth Corridor of Tanzania) community). Second, determine types of pesticides used and the proportion of women using pesticides in SAGCOT which may pose neuro-developmental effects to children. And third assessing exposure risk factors which may affect child neuro-development among women in the SAGCOT community.

## Methods

### Study area

**Fig. 1:**
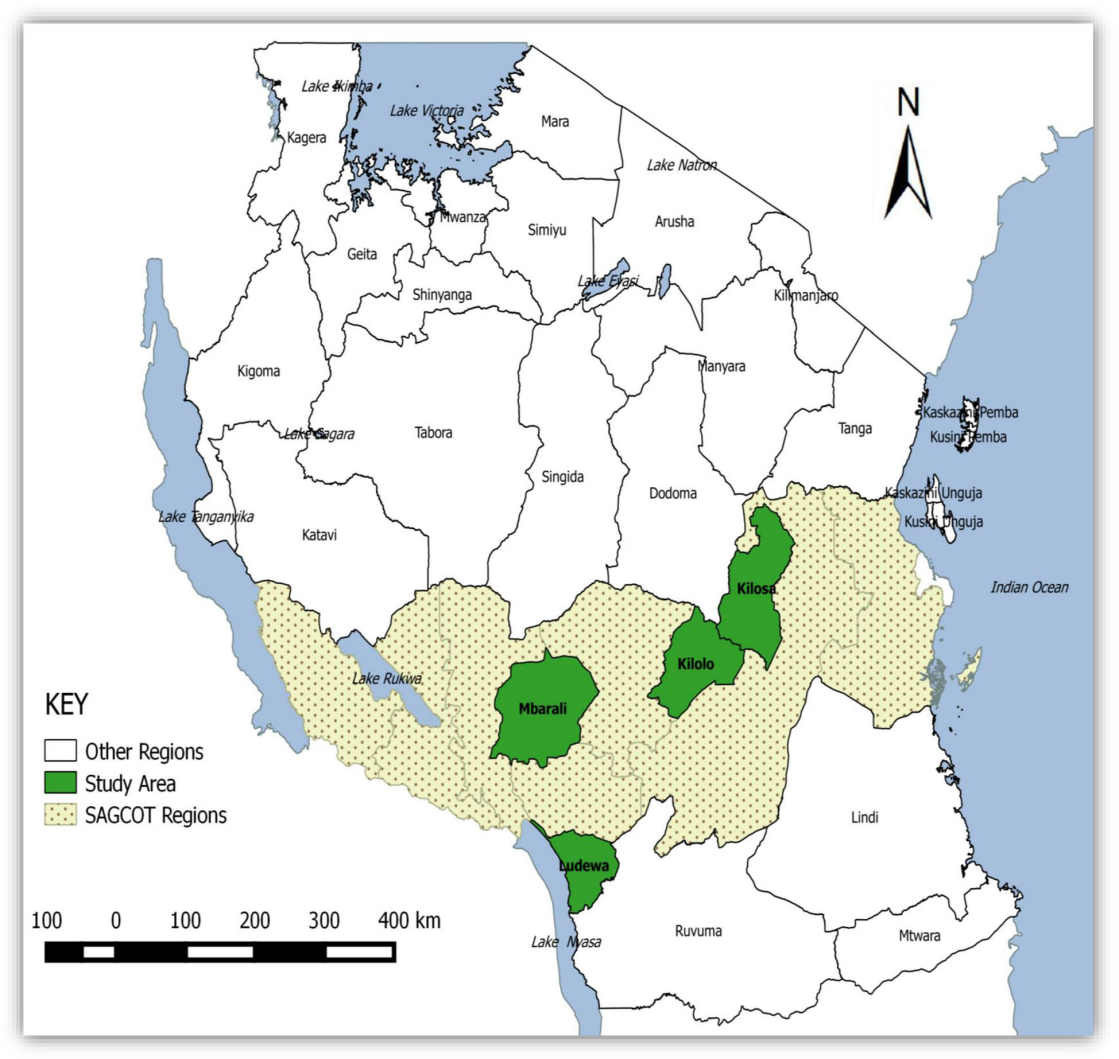
The map of Tanzania showing Southern agriculture corridor

The study was conducted in the SAGCOT corridor which is the agricultural corridor located in Southern Zone of Tanzania, covering Ruvuma, Iringa, Mbeya, Rukwa, Morogoro, Pwani and Njombe region. The agricultural corridor is characterized by high production of tomato which employs high use and reliance on pesticides thus makes the community in pesticide treadmill ultimately more prone to health effects. The Southern part of Tanzania is the most potential area for tomato production, where 50.9% of tomatoes are produced [25]. But only areas with bulk tomato production in the SAGCOT corridor were chosen in the study, as follows Iringa -Ihemi cluster, Morogoro-Kilombero cluster, Mbeya-Mbarali cluster and Njombe- Ludewa cluster. Figure 1:

### Study design and setting

A cross-sectional study of mother to child pair was conducted to assess maternal pesticide exposure and child neuro-development effect among smallholder tomato farmers in the southern corridor of Tanzania in April to July 2019. The design paved the way to assess exposure factor and the associated effects among children, namely neuro-development effect effectively since is very flexible, timely and economic [32, 33] as the research was scheduled under a period.

### Participants

The study enrolled 286 participants 286 mother [34–36] to child pair who were estimated using the Kish Leslie formula [37, 38] which is for cluster sampling whereby the proportion of children with development effect used was 40.7% referred from a study at Java in Indonesia [39]. Thus 172 of the participants were from the tomato farming community and classified as exposed and 114 of the participants were from the occupation which don’t use pesticides and classified as the Unexposed or control group. The distribution was calculated by the use of the OpenEpi calculator version 3 to check for the power of the study using the prevalence in children exposed to pesticides which were for control group assurance of study reliability, where the power was found to be 80%.

It involved children age 0–6 years found in the SAGCOT and their mothers whereby only one child found to be the youngest with the classified age was selected from each mother. Children were enrolled in an un-exposed group under the condition that their mothers were exclusively not involved in the tomato production this include Hair plaiters, teachers. and medical personnel and shoes working in shops with the exclusion of those working in pesticide shops. But children were enrolled in the exposed group if their mothers are working in tomato cultivation.

Multi-stage sampling was employed to select clusters and areas where the study was conducted. Purposive sampling was employed to select clusters, thus Ihemi, Kilombero, Ludewa, and Mbarali were selected basing on bulk tomato production. One ward from each cluster was selected by a simple random sampling method using the lottery method where names of wards were coded on a piece of paper and randomly picked.

Probability proportional to size was conducted to select three villages of interest from each of the selected wards. Thus, a list of all landing sites in the village was obtained, with their respective population of members, arranged in descending order and the cumulative population determined. The total population with the number of clusters or villages was determined, to determine the sampling interval. Then the first cluster was determined by getting a random number and the cluster that had this selected random number was the first cluster selected. The next cluster was selected by adding the sampling interval to a random number. The same procedure was repeated until all the 3 village clusters were identified plus their sampling distribution to each cluster.

The spinning method was employed to select the direction to start the selection of households and hence participants. Purposive sampling procedure was employed to sample all the mothers who were found having a child of 0–6 years at the selected village.

The main goal of purposive sampling was to focus on particular characteristics of a population of interest, which enabled the Principal Investigator to obtain answers on research questions. Sampling to the control group employed this method as well to get information of interest at the place.

The Field Mass Index (FMI) was created to incorporate both the area of any agricultural field within the designated buffer around the residence and the distance to each field from the residence (FMI= area/distance2). Therefore, the FMI was employed only at Ihemi, Mbarali, and Ludewa clusters where residential places were in proximity to the production areas**.** Where there were multiple fields within the buffer zone, the FMIs for each field were summed. Study subjects with an FMI equal to zero constituted the lowest exposed group.

### Inclusion and exclusion criteria

#### Inclusion criteria

- Children with 0–6 years old and their mothers.
- Children with their mothers who were mentally fit, defined by their ability to comprehend questions asked and answer them correctly, simply an absence of disorder of the mind
- Children who resided in a particular study area.
- Child with the lowest age to mother with more than one kid with the adhered age criterion.

#### Exclusion criteria

- Children with head trauma.
- Children with chronic illness, genetic and nutrition disorders.

#### Variables

Child Neuro-development which was the effect of exposure to pesticides measured with M-DAT whereby a child with either medium or low development in all four measured development levels of Gross, Fine, language and hearing and social was categorized as with developmental effect. Socio-demographic factors: Age, sex, residential distance, education, marital status, and average use of pesticide per year. Maternal-child related factors: Which include the following maternal factors during prenatally and at early childhood of the child: Breastfeeding, home pesticide drift, social construction during pregnancy, hygiene, staying with husband, balanced diet, pre-exposure to lead, alcohol use, place of delivering, anemic, and late delivery. Child risk factor: Child labor, play with items, crawling on the floor, eating and drinking, distance from the farm. Maternal pesticide practices factors: Training history, carrying children at farm, discomfort, financial pressure, perceived control, washing practices, gender role. Maternal occupational-related factors: Storage of utensils, Disposal of containers, use of PPEs, proximity to the farm, unsafe handling. Other factors: Which include other risk factors during pregnancy were: Delivering area, hygiene, nutrition, alcohol use, and mercury exposure.

#### Measurements

Child neuro-development was measured by the use of the Malawi child development tool (M-DAT) [40] which is the screening tool approved to be used in Tanzania for child development assessment as it is culturally appropriate [41]. The Malawi child development tool (M-DAT) Questionnaire covers four areas of child neuro-development that includes: Gross motor, fine motor, language and hearing, and social development. It involved the population of children between the ages of 0 months to 6 years old. R. A completed the questionnaire, indicating for each item “yes” if the child performs the item, or “not yet” indicating that the child does not yet perform the behavior. The tool provides results on the child neuro-development which were categorized into three categories which are; developed, medium developed, and less developed [42–44]. The assessment checklist is coded with the level of questions according to a child aged assessed. Child age was employed to determine the starting point to ask a question, thus if a child was not passing seven consecutive questions then the tool obliged the R. A to turn back to where he/she started and move back three steps if she or he fails to answer as well it obliged the R. A to further go back to other questions from which child level of neuro-development will be determined [45].

**Fig. 2:**
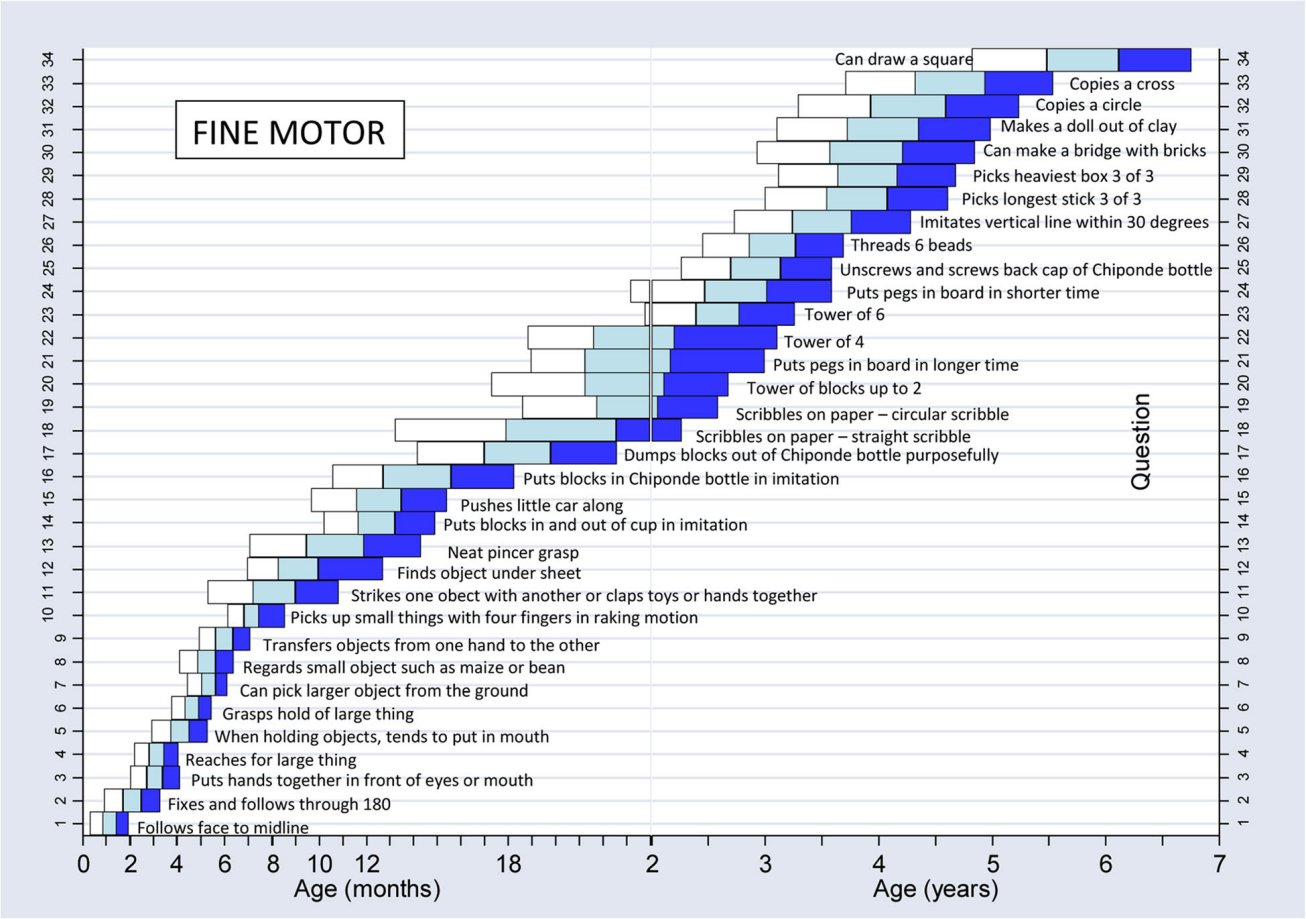
M-DAT for fine development assessment

On the grid table of obtaining the level of child neuro-development according to the question the child score, if the level obtained is on the White shaded zone indicate the child may need further assessment. Scores in the light blue shaded “monitoring” zone help identify children at risk. Professionals can give parents activities to help their child make progress in these areas before the next screening. Scores deep blue zones mean the child is doing well in these areas. The tool was developed and used in Malawi from which it was seen to be cultural perspective and acceptable thus following the study in Malawi reliability was good for items remaining with 94–100% of items scoring kappas > 0.4 for inter-observer immediate, delayed, and intra-observer testing [7, 46] Fig. 2.

WHO Anthro software was employed to account for nutrition, as it was developed with WHO Child Growth Standards in monitoring growth and motor development in individuals and populations of children up to 5 years of age [47]. The anthropometric calculator where weight and height, gender and age or month of the child were entered to obtain weight for Age (WAZ) and Length or height for age (HAZ) for a particular child from which the Z-score was obtained to each and particular child whose data were entered to the Anthro calculator. The ranges to classify the child level of Malnutrition were employed from WHO which were − 1 SD< Z< + 1 SD Normal, − 2 SD< Z< − 1 SD or 1 SD< Z< + 2 SD Mild underweight l − 3 SD< Z< − 2 SD or + 2 SD< Z< + 3 SD underweight Z<-3 SD: Z > =3 SD Severely underweight. Pre-testing of Research Tools and training to R. A was done at Temeke municipal hospital where there is a center for autism data collection and clinic to measure whether participants understand the questions.

#### Data collection

Data were collected by questionnaires by semi-structured questions. The questionnaire was employed to collect basic information on child to maternal factors and other exposure scenarios to the children as well as the demographic information. The questionnaire was developed by the author and administered to the mothers so that information can be rendered easier and collected.

Checklist was employed to assess other risk factors for the pesticide exposure to the children in the sampled areas to the SAGCOT (Southern Agricultural Growth Corridor of Tanzania) It was employed to collect information on some of the practice which accelerate the exposure to the children. Moreover the checklist assisted on the assessment of the practices regarding pesticide handling, storage use, and disposal of containers which tantamount for exposure scenarios. The recruited data collectors were trained by Muhimbili University of health and allied sciences, department of pediatric health.

### Data analysis

Descriptive analysis was done for demographic characteristics by the use of STATA to show the frequency and percentage of variables measured. Bivariate analyses (Logistic regression analysis) were conducted to examine the relationship among the factors between the independent variables and the dependent variables. Inferential analysis was employed to know which factor predicted more on the exposure to pesticides among children thus Multivariate analyses were conducted to those factors which were seen significant on the bivariate analysis. Therefore, the 95% confidence (*p*=0.05) was employed and the *p*-value was presented accordingly. To make the assessment more informative the composite variable was formed which did categorize the child development into two segments that those fully developed and those who don’t bear an acquired milestone of development as not developed (less and medium developed summed).

## Results

### Social demographic characteristics of study participants

A total of 286 study participants consented and enrolled in the study from the selected clusters in the SAGCOT zones. Most of the mother study participants were aged between 21 and 30 years with mean age (29.7, SD=5.8, and most of the children participants were female 170(59.44%) (Table 1). The majority of participants (52%, *n*=152) had attained primary education, and 70(52.6) were exposed to pesticides for more than one year (Table 2).

**Table 1:** Child Socio-demographic characteristics of study participants (*N*=286)

	Exposed		Un-Exposed		Total	
Variable	Number(n)	Percentage (%)	Number(n)	Percentage (%)	Number(n)	Percentage (%)
**Age of child in months. (Mean 2.32 Sd 1.41)**						
0–2 months	1	0.6	0	0	**1**	**0.35**
2–12 months	26	15.1	18	15.8	**44**	**15.38**
13–48 months	134	77.9	74	64.9	**208**	**72.73**
49–72 months	11	6.4	22	19.3	**33**	**11.54**
**Child Gender**						
Female	103	59.80	67	58.77	170	59.44
Male	69	40.12	47	41.23	116	40.56
**Child labor at farm**						
Yes	30	23.81	1	50	31	25
No	95	75.40	1	50	96	75

**Table 2:** Maternal Socio-demographic characteristics of study participants (*N*=286)

	Exposed		Un-exposed		Total	
Variable	Number (n)	Percentage (%)	Number (n)	Percentage (%)	Number (n)	Percentage (%)
**Age of mother in years(Mean 29.7, SD 5.8)**						
13–20	7	4.1	2	1.8	**9**	**3.2**
21–30	90	52.3	71	62.2	**161**	**56.3**
31 above	75	43.6	41	36	**116**	**40.6**
**Maternal occupation**						
Farmer	172	60			172	60
Non –farmers	144	40			114	40
**Mothers education level**						
No formal education	32	18.6	18	15.79	50	17
Primary	113	65.7	49	42.98	162	**56.64**
Secondary	23	13.37	31	27.19	54	18.88
College	4	2.33	16	14.04	20	6.99
**Field Mass Index (FMI) (Mean 1.53, SD I.64)**						
Home cultivation	173	60.49				
Yard/farm cultivation	113	39.51				
**Exposure Duration (years)**						
1	63	47				
More than 1	70	52.6				
**Cluster of dominance**						
Ihemi	66	38.37	28	24.56	94	32.9
Kilombero	49	28.49	35	30.7	84	29.4
Ludewa	27	15.7	33	28.95	48	16.8
Mbarali	30	17.44	18	15.79	60	21.0

### The level of child neurodevelopment effect

A total of 286 recruited children in the SAGCOT zone were measured by the use of M-DAT to determine their level of development. The majority of the children had a fine development effect of about 60 (20.98%) for less development and 73 (25.5%) for medium developed. The social development effect had many children with the acquisition of the milestone among the enrolled children (214, 74.8%) thus most acquired this milestone. Language and hearing were the other milestones, which most of the children did not acquire well in the area of study. Other details are summarized in Table 3 below.

**Table 3:** Child developmental effect (*n*=286)

Level of child development effect	Gross, n (%)	Fine, n (%)	Language and hearing, n (%)	Social, n (%)
Less developed	37 (12.94)	60 (20.98)	38 (13.29)	27(9.44)
Medium developed	49 (17.13)	73 (25.5)	63 (22.03)	45 (15.73)
Developed	200 (69.93)	153 (53)	185 (64.69)	214 (74.8)

The composite variable formed provide the clear picture of child level of development compared to the multiple sub category on indicated on Table 4. The composite variable was formulated by renaming less and medium developed as Not developed and the rest as well developed. Under this about 15.38% of the participants were in the category of not developed and 84.62% of the participants were in the group of well developed across all the milestones for child development.

**Table 4:** Pesticide commonly used in tomato production in the SAGCOT zone and their respective active ingredients alongside WHO toxicity class

Pesticide name.	Active ingredient	Group	WHO Toxicity class
Profecron 720EC	Profenophos	Organophosphate	II
Kungfu 720EC	Lambda-cyhalothrin 5 g/l	Pyrethroids	II
Mupaforce	Profenofos 720 EC.	Organophosphate	II
Karate 5EC	Lambda-cyhalothrin 5 g/l	Pyrethroids	II
Snow success 720	Matalaxy+ Mancozed	Carbamate	III
Ninja 5EC	Lambda-cyhalothrin 5 g/l	Pyrethroids	II
Farmerzeb 800EC	Mancozed 800EC	Carbamate	III
Linkonil 50SC	Chlorothalonil 50 g/l	Pyrethroids	III
Milthane super	Mancozed 800EC	Carbamate	III
Ivory 72 WP	Metalaxyl 80 g/kg +Mancozeb 640 g/kg	Carbamate	III
Selecron 720 EC	Profenophos 720 g/l	Organophosphate	II
Roundup	Glyphosate	Glycine Derivative	II
Fungzeb	Mancozed 800EC	Carbamate	III
Hitcel	Profenofos 400 g/l+	Organophosphate	II
Belt 480 SC	Flubendiamide 480 g/L	phthalic acid diamide	II
Imidacropid	Imidaclopid	Pyrethiod	II
Dudumectrin	Emametrin + acetameprid		
Snowcron 500SE	Profenofos	Organophosphate	II
Duduacelamectrin,	Cypermethrin	Pyrethroids	II
Link mil	Mancozeb 640 g/Kg + Metalaxyl 80 g/Kg	Carbamate	III
Duduba	Cypermethrin 100 g/l + Chlorpyrifos		II

### Types of pesticide influence child development

The composite variable formed provides a clear picture of the child level of development compared to the multiple subcategory on indicated in Table 4.The composite variable was formulated by renaming less and medium developed as not developed and the rest as well developed. Under this about 15.38% of the participants were in the category of not developed and 84.62% of the participants were in the group of well-developed across all the milestones for child development.

### Types of pesticide influence child development

Most of the study participants (94%, *n*=270) were aware of pesticide and the most prominent activity conducted by mothers at the farm was loading pesticides in preparation tanks used for mixture (51.8%, *n*=58). Although spraying was the most prominent method (81.8%, *n*=95) for pesticide application 95(81.8%) few participants spray pesticide 50 (29.79%). The most used insecticide at home is X-pel repellent spray 46(37%) and Nuvan (19%) both classified as WHO class II toxin is used in houses (Table 5).

**Table 5:** Home used insecticides

Insecticide Name	Active ingredient	WHO toxic Class	Number	Percentage
X-pel	Citrepel, Pyrethrins	II	46	37
Rungu insect killer	Permethrin	II	28	22.6
Mosquitoes mat	**DEET(**N,N-Diethyl-3methylbenzamide)	III	14	11.3
Icon	Lambda cyhalothrin	II	8	6.45
Nuvan	Dichlorvos (DDVP)	II	19	15.3
Raticide	Chlorophacinone	II	9	7.26
		Total	124	100

The most used pesticide group in the SAGCOT zone was Pyrethroids 67(31%) followed by carbamates 54(25.1%) and organophosphates 45(20.9%) which has been shown in Table 4.

Most of the participants 64(47.26%) seek advice on proper pesticides use from retailers who sell them at the shop but others seek advice from their fellow farmers 37(25.75%). Moreover, some of the tomato cultivators rely on themselves by reading the instructions and use the pesticide at farm 23 (15.75%).

### Factors for child development

#### Social demographic factors as related to child development

On bivariate analysis model, it was reported that the odds of having a child with development effect were 4.3 times those worked more than a year as compared to those working for less than a year OR 4.3 (1.5–11.5). Mother living in the Ludewa cluster were more likely to have a child with a developmental effect compared to those mothers living in Ihemi cluster OR=11.9 (2.7–52.07) in the SAGCOT area. Thus cluster dominance and time of pesticide exposure were significant predictors of developmental effect.

#### Maternal related factors compared to child development effect

On bivariate analysis, the maternal related factors show that the odds of a child having development effect was lower, about 0.2 among children to those mothers received advice on the proper use of pesticide from retailer OR = 0.2 (0.061–0.81) as compared to those seeking themselves. The odd of a child having development effect was higher, about 4.49 among children whose mothers sought advice on proper storage from neighbor OR =4.49 (1.2–16.4) as compared to those who sought advice from retailers. Those mothers who applied pesticide by burning method had their children with lower odds 0.3 of having development effect or not acquiring developmental milestone (OR=0.32(0.1–0.9) in comparison to those who employed spraying methods. However, the odds of the child whose mother had not received any training regarding pesticide management and proper use was 3 times higher compared to those who received such training OR= 3 (1.9–4.7). The odd of the participants who were residing more than 5 km from the farm, 4.3 times higher to have their children with development effect as compared to those who resided less than 5 km near the farm (OR =4.3(1.5–11.5) (Table 6).

**Table 6:** Relationship between maternal related factors and child development effect

		Development effect		Crude Odd Ratio (95% CI)	***P***-value
Variable	Total number	Developed n(%)	Not developed n(%)		
**Advice on proper use**					
Consultancy	5	1 (73.33)	4 (26.67)	0.24 (0.04–1.25)	
Neighbour	37	34 (91.89)	3 (8.11)	9.2 (0.96–11.2)	
Herself	25	23 (92)	2 (8)	ref	
Retailers	69	48 (71.64)	19 (28.4)	0.2 (0.061–0.81)	**0.029**
**Advice on proper storage**					
Consultancy	15	11 (73.33)	4 (26.67)	1.8 (0.3–3.8)	
Neighbour	38	34 (91.89)	4 (10.81)	4.49 (1.2–16.4)	
Herself	24	23 (92)	1 (4.35)	4.6 (0.98–21.2)	
Retailers	69	48 (71.8)	19 (26.1)	ref	**0.042**
**Method of application**					
Spraying	44	39 (88.64)	5 (11.36)	Ref	
Burning	52	37 (71.15)	15 (28.9)	0.32 (0.1–0.9)	**0.039**
Smearing	7	7 (100)	0		
**Distance to farm in km**					
**< 5**	110	75 (66.98)	35 (33.2)	Ref	
**5 and above**	26	17 (95)	9 (5)	4.3 (1.5–11.5)	**0.000**
**Time of exposure to the pesticide in year**					
1	2	1 (50)	1 (50)	Ref	
More than 1	120	102 (85)	18 (15)	5.7 (0.34–94.75)	0.176
**Received training on pesticide**					
**Yes**	24	24 (100)	0)	Ref	
**No**	106	80 (75.47)	26 (24.53)	3 (1.9–4.7)	**0.007**

#### Maternal related practice factors

Maternal practice factors were the predictor mother’s practices which accelerate maximum exposure to the child thus on bivariate analysis model the odd of the child having development effect was higher, about 5.8 to mothers who worked while pregnant as compared to those mothers who were not working while pregnant OR=5.8(1.29–26.3).

#### Nutritional, and relative activities factors

The odds of the child to have development effect was higher 3 times to mother who were taking alcohol as compared to those mothers who were taking alcohol OR= 3(1.03–8.7). And the odd of a child having a developmental effect on the underweight child was 2.5 times as compared to normal weighted child OR = 2.5(1.24–5.16) on the bivariate analysis model (Table 7).

**Table 7:** Nutrition and related activities factors

		Child Development effect			
Variable	Total Number	Developed, n (%)	not developed, n (%)	Crude Odd Ratio (95% CI)	***P***-value
**Weight for age**					
Normal	156	124(79.5%)	32(20.5%)	ref	
underweight	130	118 (90.8%)	12(9.23%)	2.5(1.24–5.16)	**0.01**
**Husband work**					
Barber/driver/Mechanical	87	74(85)	13(14.9)	1.11(0.53–2.31)	0.362
Doctor/nurse.	15	15(100)	0(0)		
Office/teacher	37	30(81)	7(18.9)	ref	
Farmer and not working	149	123(83.6)	24(15.38)		
**Severe malaria**					
**Y**es	38	34(89.47)	4(10.53)	ref	
No	248	218(87.9)	30(121.1)	1.2 (0.5–3.14)	0.577
**Drinking alcohol**					
Yes	60	56(93.3)	4(6.67)	3(1.03–8.7)	
No	228	186(82.3)	40(17.70)	ref	**0.035**
**Smoking**					
Yes		24(85.7)	4(14.29)	ref	
No		218(84.5)	40(15.5)	0.9(0.3–2.7)	0.865

#### Multivariate logistic regression

On multivariate analysis using binary logistic regression, after adjusting for age of the mother the factors, pointed that those who stayed 5 km and above from their farm were 9 times risky to have their child with development effect [AOR= 9.4(4.2–20.5)] compared to those staying below 5 km. The odds of those who work for more than one year at the farm were higher 4.26 as compared to those who worked less than one year [AOR 4.26(1.6–12)]. The odds of having a child with a development effect was 5.8 times higher to those who work while pregnant as compared to those who are working while not pregnant [AOR=5.8(1.29–26.3)] (Table 8). Other variables were removed due to collinearity effect they cause such as advice on the use and advice on the storage.

**Table 8:**
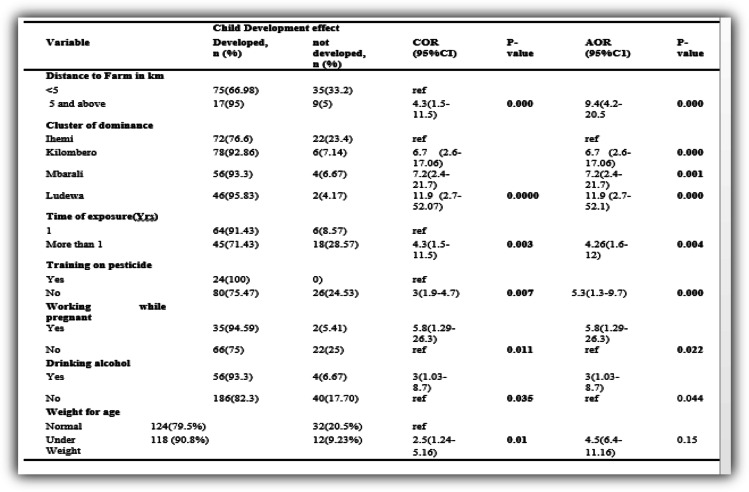
Bivariate and multivariate logistic regression for the factors independently associated child development among mother

## Discussion

### Potential study limitations and delimitation

Most of the information obtained from the study was based on self-report, hence subject to the information and recall bias as the study relied on the reported information regarding maternal exposure. It is more likely that the respondents might have provided wrong information for different reasons such as personal interest or fear of disclosing information. This challenge was mitigated by triangulating information from different sources such as observation. The theme of the study was explained clearly until understood before a respondent gave consent.

More effective measurement is recommended that enough time needs to be spent in measurement to get attached to the child. The study did not consider intensively the genetic factors and exposure dose which might be an important predictor of the child development effect in a precise manner. This aspect requires another intensive study to explore and assess.

The cross-sectional nature of the study allows inference but does not establish a causal relationship. Despite these limitations, this study contributes immensely to the understanding of child development effect, associated maternal occupation, and behaviors of the smallholder’s tomato cultivators in the SAGCOT region, where most of the participants practice intensive farming that uses a lot of pesticides.

The recall bias was solved by carefully defining the research question that was ambiguous to make the sampled population aware of the information required. Also, the participants who had not been engaged in tomato farming for more than 10 years were removed from the study. The researcher’s assistants undergo a short training on how to calculate the scores for child development and testing on the tool to keep effective measures. All the independent risk factors were controlled on the exclusion criteria to remove some of the ambiguities that might arise as a result of underestimating them.

### Magnitude of children with developmental effects among women

The study by Lekei et al. [48] elaborates that most of the farmers are not aware of pesticides, especially on proper uses mostly those educated ones but this report has pointed out that regardless of their educational level most of the study participants were aware of pesticide. This has been contributed by their organization formed in the SAGCOT zone according to their clusters such as the Lugelele scheme irrigation community, Ilula SACCOS, which also provide knowledge to farmers on pesticide. Kumari et al. [49] report in India pointed out that knowing does not make farmers practice safe handling of pesticides as, from 80% of farmers with knowledge, only 50% were observed practicing safety measures while using pesticides [50–53]. This portrayed the same under the SAGCOT zone, where most know but few practiced safe handling.

The report pointed out that the most prominent activity conducted by women at the farm was loading pesticides in preparation tanks used for mixture 58(51.8%) though spraying was the most prominent method for pesticide application 95(81.8%). A few of mothers sprayed pesticide 50 (29.79%). This aligned with Mrema et al. [54] and Karith [70] reports pointed that.... report which pointed out that women in developing countries have taken on additional agricultural roles that were initially perceived to be men’s work. These include mixing and applying pesticides in agriculture. Though in the SAGCOT zone women role at the farm is harvesting and uprooting only, economic constraints and the SAGCOT policy which seek to increase production act as the pull factor for women to work in tomato cultivation. They don’t opt for the type of activity thus are made captive under pesticide exposure hence this account for pesticides drift to their child.

The Mount Sinai Children’s Environmental Health Center report pointed out the effect on indoor use of polychlorinated biphenyls on pregnancy outcome and child neuro-development in an inner-city multiethnic [24]. EPA [7] has reported that as much as 75% of all household pesticide use occurs inside the home and 22% occurs in yards and gardens surrounding the home [7]. This report found that X-pel and Rungu insect killers were the most insecticides used at home that entered the class II of WHO toxic classification. Though weather variability and climate change, most farmers in the SAGCOT area were forced to use this insecticide in their homes, which posed an additive effect.

The most used pesticides (59%) were from the class II of the WHO classification This was in agreement with Lekei et al. [48] report which pointed specific active ingredients associated with poisoning in this study, OP’s (42.4%) and class II agents (77.6%) accounted for the highest proportions. At the SAGCOT area, the use of class II was a bit low due to their habit where most of the farmers mixed pesticides of various classes to improve the vigor. This limited them to use more of class II pesticides but also these two studies were conducted under variety perspective one on tomato and the later on vegetable and horticulture community at Arumeru.

A study by Lekei et al. [48] report the active ingredients most commonly reported by farmers which were Mancozeb (80%), Profenofos (72%), Chlorpyrifos (48%),) in Arumeru dc. Study by Mtashobya [25] to tomato grower in Mazombe, Irole and Lulanzi in Kilolo report 10 different active ingredients chlorothalonil and metalaxyl/mancozeb products based formulations were more commonly mentioned both contributing 38% of all products accessible to farmers followed by lambda-cyhalothrin and profenophos (28%), mancozeb (9%), endosulfan, chlorpyrifos, dimethoate, triadmenol and triadimefon at 5% each.

This report has found out that the most common ingredient used at the SAGCOT place, [55] Kilolo inclusive, was the same as those of Mtashobya et al. [25] pointed but this report came out with glyphosate, flubendiamide and imidacloprid, which were not reported.

The most used pesticide group in the SAGCOT zone was the pyrethroids group (31%, *n*=67), followed by carbamate (25.1%, *n*=54) and organophosphate (20.9%, *n*=45), which both posed neurodevelopment effect to the children. The variation in the type of active ingredient used was mostly due to the operation, especially in the SAGCOT whereby farmers, through their organization agreed on which pesticide to use most and also the type of pest to be controlled.

Lekei et al. [54] study reported that many retailers were not well trained, a fact that draws attention on the information landed to the farmers if at all was effective or not. Some studies pointed out that a good number of smallholder vegetable farmers (58.7%) do not have access to information on safety tips about pesticide handling or training on pesticide management as per study in Nigeria by Ugwu et al. [56]. Lahr’s [32] study pointed that with SAGCOT vision, there was an upsurge of risk to agricultural workers through inappropriate use of pesticides [57].

That was pinpointed on the aspect of pesticide use at the SAGCOT. This information is useful in guiding focused intervention towards the achievement of self-environmental and health for all and this will ultimately make the society reach the ladder of good Health and Well-being as per SDG 2015–2030.

### Risk factors for child neuro-development effect

Socio-demographic characteristics that were significantly associated with child neuro-development effects were a cluster where a participant stays and duration of work. Thus the odds were 4.3 times to those who worked more than a year at the farm as compared to those who worked for less than one year. This aligns with the study in Washington [58, 59], and that of Mark [60] which both pointed exposure time of more than one has a potential effect on the individual at the farm using pesticide without proper management. So limiting or control of the exposure duration among female workers in the farm has a potential risk reduction.

The child whose mother lived in the Ludewa cluster was more likely to have neurodevelopmental effects compared to those children living in the Ihemi cluster in the SAGCOT area. This aligns with Handal et al. [59] who pointed out that maternal occupation in the cut-flower proximity to place of residence where there was a lot of pesticide use with the non-nomadic population, pesticides effects were more pronounced as in Ludewa where the population was not nomadic and the use of pesticide was high. This portrays that to the participants who are non-nomadic and they conduct tomato cultivation near there residency the effect of pesticide to their child is more pronounced so denouncing home cultivation to non-nomadic cultivators will help to control the effect.

Maternal-related factors that were found to be significantly associated with child neurodevelopment effects were a distance from the farm and area, advice-seeking on the proper usage of pesticide and proper storage of pesticide, training history on pesticide usage, and method of application of pesticide. The odd of a child having development effect was lower about 0.2 among children whose mothers received advice on the proper use of pesticides from retailers as compared to those who relied on themselves for advice. The odd of a child having development effect was higher, about 4.49 among children whose mother sought advice on proper storage from neighbors as compared to those who sought advice from retailers. A study by Liu [6, 61] and Eskanazi [62, 63] both align with the findings by pointing out that most farmers sought advice from retailers. The challenge with our perspective is their low education which the majority of them have that most of them did not know how to read and write, the reason why seeking advice from retailers was a bit protective but from themselves as a risk factor. Also, receiving training regarding proper management of pesticide was mentioned as the risk factor, whereby to the study most of the farmers did not receive as mothers had not received training regarding pesticide management and proper use were 3 times higher likelihood to have a child with development effect. Those mothers who apply pesticide by burning method had their child with lower odds 0.3 of having development effect this portrays that burning is protective which is true as most of the study pointed spraying as the means for exposure Mrema et al. [49] Eskanazi [64–66] and Handal et al. [59].

This report revealed that the odd of participants residing more than 5 km and above from the farm were 4.3 much higher to have their child with neurodevelopment effect as compared to those who reside less than 5 km which differs from many studies as Handel et al. [67] and Liu [6] both describe on proximity to pesticide sprayed area as a risk factor for exposure and health effect. The variation observed is due to the nature of the participants in the SAGCOT thus most of the tomato cultivators do not stay near their farm, since these areas are infringe with public serves like electricity and amenities thus they store their pesticides and mix plus packing in the sprayer tank at home to reduce some of the tasks while at the farm also they carry already mixed pesticide to a long distance from their home to farm ultimately they go to the farm while already stained and exposed to pesticides more at home this makes child at home above 5 km to be at risk of the neuro-development effect.

Working while pregnant was a factor that indicated the association with the child neuro-development levels. The odd of the child having development effect was higher, about 5.8 that of mothers working while pregnant as compared to those mothers who were not working while pregnant. Thus, working while pregnant was a risk factor for child development. This was in line with the Autism Society findings in California that exposure to pesticides during pregnancy increases a child’s risk of autism by nearly 10% [68, 69]. Since there is an income finding struggle to refrain from poverty, most women were compelled to work while pregnant in the SAGCOT area.

The study considers all the potential confounders which might affect child neuro-development such as mercury exposure, maternal alcohol and cigarette taking, delivery period at place, nutrition, and family history. From which none of the factors had a potential significant effect but only nutrition seems to be the effect modifier as the adjusted Odd AOR=7.8(1.29–36.3) when analyzed with working while pregnancy. Thus possibly pesticides interact with the nutrient intake of the child and ultimately less developed.

### Recommendation

The neurodevelopment component on a child is very vital so as to have a future which is health, thus prenatally and early childhood pesticides exposure should be halted with much effort and concern. As the high prevalence of child development effect in the SAGCOT area among the tomato cultivators is an indication that pesticides pose an effect to the zone. So it is recommended that the health promotion component should be incorporated in the training as we have seen most of the community in the SAGCOT area has not received any training on the proper use except for the Ludewa cluster where they have their organization scheme which provides education.

Custodian of managing and controlling pesticide at farm and home varies in the Tanzania, as the Ministry of Agriculture is for farm pesticide management and the Ministry of health is for home or indoor pesticide management this makes the management not effective since the same pesticides are managed under different custodians with their varying locality of use. So we recommend the custodian for management of all pesticides should be rendered to one ministry whereby TPRI should be given its authority as it was before the enacting of the Plant Protection Act.

This report has pointed the weak surveillance which is dominating in Tanzania regarding pesticide exposure and its associated effect. The need for more studies and improvement in the areas of surveillance is of necessity such as to empower TPRI with more resources being human under health-related or other to make surveillance improved. Surveillance system regarding pesticide health effects should be conducted especially to the vulnerable population such as children and women while they are pregnancy this will lead to the creation of a healthier society and brighter future to the young generation which is free from pesticide-related health effects.

There is a pressing need to draw attention to the policymakers on the need to enact the pesticide policy in Tanzania and increase resources in various areas. The need for more extension officers and more studies on adverse effects related to pesticides in exposed agricultural areas such as the Ihemi cluster and Ludewa are significant. The Study can be taken to the government for policymaking on the adverse effect of pesticide and implementation measures.

## Conclusion

The findings from this study have indicated that maternal pesticide exposure has a potential association with child neurodevelopment among farmworker residents in the SAGCOT area. These findings are not oriented to denounce farmers with the use of pesticides but to channel a trade-off between economic struggling with their health and their beloved hence to refrain them from pesticide treadmill or captivities, which accelerate them to have health effects. The study has shown that the factors that were significantly associated with child development among women in the SAGCOT zone were the distance from the farm, cluster where the participant resides, advice on proper use and storage, training acquisition, alcohol use, working while pregnant and duration of working at the farm which advocate on the prenatally and early childhood neurodevelopment infringement**.** The genetic factor on the child neuro-development was not considered since the study was a cross-sectional design and limited with resources like time. Our findings have provided strong baseline data for planners and implementers if taken into consideration to eradicate poor management of pesticides.

## Supplementary Information

**Additional file 1.** Questionnaire.

## Data Availability

The data sets used and analyzed during the current study are available and still under analysis for subsequent publications but will be available upon request from the correspond author.

## Abbreviations

ACHE: Acetylcholinesterase
ADHD: Attention-Deficit/Hyperactivity Disorder
ASD: Autism Spectrum Disorder
ASQ-3: Age and Stage Questionnaire third edition
CHAMACO: Center for Health Assessment of Mothers and Child organization
CP: Cerebral Palsy
EPA: Environmental Protection Agency
M-DAT: Malawi child development assessment tool
NRC: National Research Council
OP: Organophosphate
PI: Principal Investigator
PBA-3: 3-phenoxybenzoic acid
PPE: Personal Protective equipment
RAs: Research Assistants
SAGCOT: Southern Agricultural Growth Corridor of Tanzania
SDGs: Sustainable Development Goals
TCP: 3,5,6-Trichloro-2-Pyridinol
TPRI: Tropical Pesticide Research Institute
WHO: World Health Organization
MEVT: Ministry of Education and Vocational Training
