# Supplementary material for: Maternal pesticide exposure and child neuro-development among smallholder tomato farmers in the southern corridor of Tanzania
Source: BMC Public Health. 2021 Jan 20;21:171. doi: 10.1186/s12889-020-10097-6 (PMC7818734; doi:10.1186/s12889-020-10097-6)
Supplement: Supplementary file 1 — Additional file 1. Questionnaire. [file 12889_2020_10097_MOESM1_ESM.docx]

**Additional file 1**: Questionnaire.

This is the tool which was used for data collection developed by the corresponding author.

**Introduction**: This is the tool which is oriented to collect information on mother to child pesticides exposure so as to assess the effects of pesticides to the child which is neurodevelopment in particular, the answers to these questions will give answers to the research study titled below.

**TITLE**: Maternal pesticides exposure and child development among smallholder tomato farmers in southern corridor of Tanzania**.**

**PART ONE: Social demographic characteristic**

Serial number ………

Date…….………………….. Phone number (Mother) Optional…………………

1. Name of the cluster …………….. 2. Name of the village ……………………….

3. Age (Mother)……………. 4. Occupation (Mother)………………….

1. How many children do you have………………
2. What is their age

| S/N | Age | **Delivering terms** | | Special health condition | Birth date |
| --- | --- | --- | --- | --- | --- |
|  |  | Full Gestation | Not full gestation |  |  |
|  |  |  |  |  |  |

7. Have you ever lost any child (if No go to qn 10)

a. Yes b .No

8. If yes to question 7 ,how many ………………………………

9. If yes to question 7, what Happen……………………………………………

1. Race (mother)
   1. African b .Arabic c. Asian d. others

11 what is your earning per month (mention) …………………………….

12 .Educational level (mother)

1. No formal **b**. Primary **c**.Secondary **d**.College/university **e**.No response

**13.** Marital status.

**a.** Married **b**. Single **c**. Divorced **d**. Widowed e. Cohabiting

14. Is your husband supporting you in child care?

a. Yes b . No

15. What kind of support does he give?

………………………………………………………………………………………….

………………………………………………………………………………………….

**16.** What activities do you do in tomato cultivation?

**a.** cultivating b. harvesting and uprooting c spraying pesticides d. Mixing pesticides

17. What is the approximately distance from the farm you work in to your home… …K/Meters

**PART TWO: Pesticides use and their types.**

1. How do you control pests or weeds? Fill the table below.

|  | **Area of consideration** | **Pesticides name** | **Product mixed** | **Method of control** |
| --- | --- | --- | --- | --- |
| 1 | At home |  |  |  |
| 2 | At the farm |  |  |  |
| 3 | At the garden |  |  |  |

2 .Do you know pesticides? If No Skip Question 4.

a. Yes b .Sometime c. No

3. Have you ever personally conducted the following?

| S/N | ACTION | MARK (tick) | When did you start |
| --- | --- | --- | --- |
| 1 | Mix pesticides |  |  |
| 2 | Load pesticides |  |  |
| 3 | water refilling in Tanks |  |  |
| 4 | Refilling pesticides in sprayer |  |  |
| 5 | Spraying pesticides |  |  |

4. Which type of pesticides do you usually handle (Mention them)

| S/N | Types | Mix | Mixing method used | Spray | Spraying method |
| --- | --- | --- | --- | --- | --- |
|  |  |  |  |  |  |

6. Do you wear protective clothing when handling pesticides?

**a**. Yes b. No

7.if yes to question 6 ,what kind (mention)

| Kind | Using frequency | |
| --- | --- | --- |
|  | Always | sometimes |
|  |  |  |

8. Who advice you on the right way to use of pesticides?

**a**. From retailer **b.** From consultancy services **c**. From neighbors

**d.** Others (please specify)……………………

9. Who advice you on the right way to store pesticides?

**a**. From retailer **b.** From consultancy services **c**. From neighbors

**d.** Others (please specify)……………………

. Yes b. No c. sometimes

**10.** Do you use alternative to synthetic pesticides?

**a**. Yes b. No

11. If yes to qn 10 which alternatives

| Name | Always | Sometimes. |
| --- | --- | --- |
|  |  |  |

12.What is your source of drinking water …………….

**13.** How far is your drinking water from the farm? (Mention)…….. Meter

**PART THREE : Risk factors for child development effect.**

| Pesticides Exposure Risk Factors. (Circle the answer.) |
| --- |
| 1.With reference to qn 4 part 1.How many years have you been working………….…yr/month  2. How soon do you change clothes after work?  a. Immediately b.lately  3. Where do you dispose pesticides empty container?   1. At farm b. At home yard c. Haphazardly   4. Where do you dispose pesticides left over?  a.At farm b. At home yard c. Haphazardly  5. Do you go with your child at farm while working?  a. yes b.No  6. Is your child working in farm field?   1. Yes b. No   7.If yes to qn 6 is she or he working  a. always b. sometime  8. Where you working at your working place during pregnancy?   1. Yes b. No   9. Have you received any training on pesticides management?   1. Yes b. No   10. Where do you wash pesticides equipment used at farm?  a. At home b. Bathroom in the house c. Outside the yard  d. Nearby River/ e. Other  11. Where do you store the pesticides?   1. At farm b. home c. any place   12. What is your husband occupation ………………….  13. Do you wash working clothes for your husband as he comes from the working field? a.Yes b. No  14. Have you ever breastfed your child while working at farm?   1. Yes b. No   15. Do you eat while at the cultivation area?   1. Yes b. No   16.For how long do you work at farm ………………..per day |

| Other Exposure Risk Factors. (Circle the answer.) |
| --- |
| 1. Does your child ever suffer from severe malaria?   1. Yes b. No   2. Is the family provided with Latrine facilities?   1. Yes b. No   3. Are you drinking alcohol?  a.Yes b. No  4. Have you ever worked in the mining areas?   1. Yes b. No   5. Are you smoking cigarette   1. Yes b. No   6.Have you experienced any injury while at farm.(if yes go to qn 7  a.Yes b. No  7. Do you think it was due to pesticide?  a.Yes b. No c. don’t know  8.Have you ever been anemic while pregnancy   1. Yes b. No   9. Where do you deliver your children………………………  10. Have you ever gone there lately for delivering?   1. Yes b. No |
